# Mars’s induced magnetosphere can degenerate

**DOI:** 10.1038/s41586-024-07959-z

**Published:** 2024-09-18

**Authors:** Qi Zhang, Stas Barabash, Mats Holmstrom, Xiao-dong Wang, Yoshifumi Futaana, Christopher M. Fowler, Robin Ramstad, Hans Nilsson

**Affiliations:** 1 Swedish Institute of Space Physics, Kiruna, Sweden; 2 Department of Physics, Umeå University, Umeå, Sweden; 3 Department of Physics and Astronomy, West Virginia University, Morgantown, WV USA; 4 Laboratory for Atmospheric and Space Physics, University of Colorado, Boulder, CO USA

**Keywords:** Magnetospheric physics, Exoplanets

## Abstract

The interaction between planets and stellar winds can lead to atmospheric loss and is, thus, important for the evolution of planetary atmospheres^1^. The planets in our Solar System typically interact with the solar wind, whose velocity is at a large angle to the embedded stellar magnetic field. For planets without an intrinsic magnetic field, this interaction creates an induced magnetosphere and a bow shock in front of the planet^2^. However, when the angle between the solar wind velocity and the solar wind magnetic field (cone angle) is small, the interaction is very different^3^. Here we show that when the cone angle is small at Mars, the induced magnetosphere degenerates. There is no shock on the dayside, only weak flank shocks. A cross-flow plume appears and the ambipolar field drives planetary ions upstream. Hybrid simulations with a 4° cone angle show agreement with observations by the Mars Atmosphere and Volatile Evolution mission^4^ and Mars Express^5^. Degenerate, induced magnetospheres are complex and not yet explored objects. It remains to be studied what the secondary effects are on processes like atmospheric loss through ion escape.

## Abstract (web summary)

When the cone angle between the solar wind velocity and the solar wind magnetic field is small at Mars, the induced magnetosphere degenerates.

## Main

The focus of this case study on a 4° cone angle is to describe the global structure of a degenerate, induced magnetosphere and to identify the main domains and regions. First, we found good agreement between the hybrid simulations and measurements by both the Mars Atmosphere and Volatile Evolution (MAVEN) mission and Mars Express (MEX) (see Methods for details). This enabled us to broaden the investigation of this degenerate, induced magnetosphere to encompass various parameters across the entire vicinity of Mars, beyond observed quantities along the trajectories of the observing spacecraft. We now present some essential parameters from hybrid plasma simulations in a Mars–Sun–electric field (MSE) frame (see Methods for details of the simulations), including the magnetic field and the densities of primary ions, namely, protons and ${{\rm{O}}}_{2}^{+}$.

**Fig. 1:**
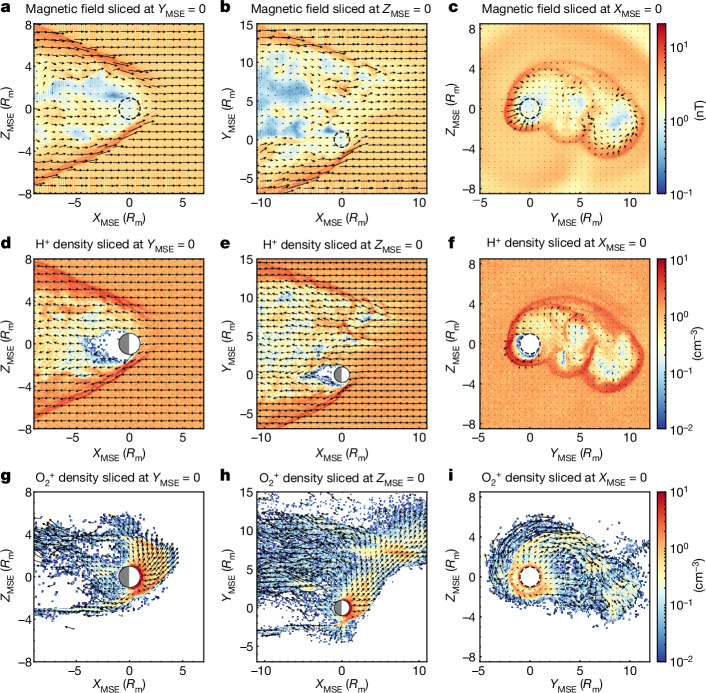
Simulation results. **a**–**c**, Magnetic field strength and direction projected in the plane shown: *Y*_MSE_ = 0 (**a**), *Z*_MSE_ = 0 (**b**) and *X*_MSE_ = 0 (**c**). **d**–**f**, Proton density: *Y*_MSE_ = 0 (**c**), *Z*_MSE_ = 0 (**d**) and *X*_MSE_ = 0 (**e**). **g**–**i**, ${{\rm{O}}}_{2}^{+}$ density: *Y*_MSE_ = 0 (**g**), *Z*_MSE_ = 0 (**h**) and *X*_MSE_ = 0 (**i**). The black arrows represent the direction of ion flow. Axes are scaled by the Mars radius, *R*_m_.

**Fig. 2:**
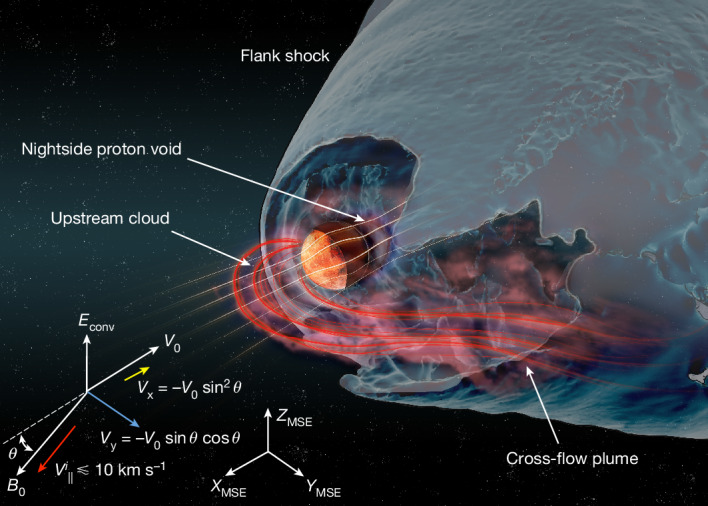
Artist’s impression. Main domains and boundaries of the degenerate magnetosphere and the near-Mars environment for a near-parallel IMF. MSE coordinate axes and key vectors are shown for reference (not to scale). See text for notation.

Figure 1a–c displays the magnetic field magnitude and magnetic field vectors projected on all three orthogonal planes. Key features include: (1) the absence of an increase in the magnetic field indicative of a bow shock on the dayside (Fig. 1a,b), (2) the presence of a shock-like structure at the flanks (Fig. 1a,b), (3) a highly asymmetric structure extending towards the +*Y*_MSE_ axis (Fig. 1b,c). Similar structures are evident in the density plots for both presented populations (Fig. 1d–i). No signatures of an increase in the magnetic field at low altitudes are visible. Indeed, an induced magnetosphere was not generated, and thus, no bow shock appeared on the dayside, although a clear boundary of unknown nature is distinguishable at the flank. Additionally, there were significant fluctuations in the wake region. In the *Y*_MSE_–*Z*_MSE_ plane (Fig. 1c), an extension of the shock-like multi-structure emerges as a consequence of the movement of different planetary ion species.

Figure 1d–f shows the proton density distribution. The solar wind propagated all the way down to the inner boundary and precipitated onto the exobase (Fig. 1d,e). There was no discernible increase in the proton density on the dayside that could be indicative of a magnetosheath. Furthermore, there was no reduction in the proton density near the planet, particularly in the region of ionospheric heavy ions (Fig. 1g,h). No induced magnetosphere formed.

In Fig. 1g–i, we display the ${{\rm{O}}}_{2}^{+}$ densities. The interaction region was significantly larger (more than ten times the volume) than for a normal interplanetary magnetic field (IMF)^6^. Planetary ions propagated far upstream along the magnetic field lines and also travelled over approximately ten Mars radii perpendicular to the solar wind direction, forming a cross-flow plume (Fig. 1h,i). The ion dynamics was defined by the ambipolar field close to the planet, where the gradients of the electron density were the strongest, and by the weak convective field when ions reached altitudes of hundreds of kilometres and the ambipolar field vanished. These heavy ions initially moved along the +*Z*_MSE_ axis (**E** direction, −**V** × **B**) and subsequently drifted along the +*Y*_MSE_ axis (**E** × **B**). The trajectories depended on the ion species and their Larmor radius.

The simulations presented here provide insights for comprehending the electrodynamics of a degenerate, induced magnetosphere. We begin with simple estimates of the electric fields that define particle motion close to Mars. The total electric field is the sum of the convective electric field **E**_conv_, the Hall field **E**_Hall_ and the ambipolar field **E**_amb_ (generalized Ohm’s law):

1 $${\bf{E}}={{\bf{E}}}_{{\rm{conv}}}+{{\bf{E}}}_{{\rm{Hall}}}+{{\bf{E}}}_{{\rm{amb}}}=-\,{\bf{V}}\times {\bf{B}}+\frac{{\bf{J}}\times {\bf{B}}}{{n}_{{\rm{e}}}e}-\frac{\nabla {p}_{{\rm{e}}}}{{n}_{{\rm{e}}}e},$$

where **V** is the local plasma velocity, **B** is the magnetic field, **J** = ∇ × **B**/*μ*_0_ is the current density, *p*_e_ is the electron pressure, *μ*_0_ is the magnetic constant, *e* is the elementary charge, and *n*_e_ is the electron density, which was equal to the ion density.

For our rough estimations, we assumed that only *E*_conv_ depends on the cone angle as ${E}_{{\rm{conv}}}\approx VB\,\sin \theta$. Therefore, there should be a critical cone angle *θ*_cr_ when the convective field becomes comparable to either the Hall field *E*_conv_ ≈ *E*_Hall_ or the ambipolar field *E*_conv_ ≈ *E*_amb_. The Hall term can be estimated as

2 $${{E}}_{{\rm{Hall}}}\approx \frac{{{B}}_{0}^{2}}{L}\frac{1}{4{\rm{\pi }}{n}_{{\rm{e}}}e}$$

and the critical cone angle as

3 $$\sin {\theta }_{{\rm{cr}}}\approx \frac{{{B}}_{0}}{L}\frac{1}{4{\rm{\pi }}{n}_{{\rm{e}}}e{V}_{0}},$$

where *B*_0_ = 2 nT is the typical magnetic field strength, *L* ≈ 3,400 km (about the Martian radius) is the typical size of the magnetic field change, *V*_0_ = 400 km s^−1^ is the typical solar wind velocity and *n*_e_ = 10^3^ cm^−3^ the typical ionospheric density at a height of several hundred kilometres. The critical angle given by equation (3) is non-physically small, less than 0.001°, and thus, the Hall electric field does not define the plasma dynamics at low altitudes away from crustal fields.

The ambipolar field *E*_amb_ can be estimated as

4 $${E}_{{\rm{a}}{\rm{m}}{\rm{b}}}=\frac{{\rm{\nabla }}{p}_{e}}{e{n}_{{\rm{e}}}}\approx \frac{{P}_{{\rm{d}}{\rm{y}}{\rm{n}}}}{e{n}_{{\rm{e}}}H},$$

where the solar wind dynamical pressure *P*_dyn_ ≈ 0.3 nPa must be balanced by the ionospheric pressure, *n*_e_ ≈ 10^2^–10^3^ cm^−3^ is the typical ionospheric density where the interaction takes place and *H* ≈ 100 km is the typical scale height. The critical angle *θ*_cr_

5 $$\sin {\theta }_{{\rm{cr}}}=\frac{{E}_{{\rm{amb}}}}{{E}_{{\rm{conv}}}}$$

is around 1°–10° (close to the one observed). The ambipolar field defines the plasma dynamics near the planet for a degenerate, induced magnetosphere.

In our model, we did not observe any increase in the magnetic field to balance the solar wind dynamic pressure or the formation of any magnetic barrier. Consequently, no bow shock formed on the dayside, and, probably, only relatively weak quasi-perpendicular shocks formed on the flanks, but this subject is for future studies. Owing to the finite conductivity, the IMF diffused into the ionosphere and reached low altitudes to envelope the obstacle. In reality, solar wind protons on these magnetic field lines interact collisionally with the atmosphere and ionosphere and get lost, which depletes the magnetic tubes. The hybrid model is collisionless but the protons still became lost when impacting the simulation inner boundary. This resulted in the formation of a proton void behind the planet (Fig. 1d–f). This unique void differs from those in nominal cases, which arise not from proton deflection after encountering an obstacle but from collisional interactions within the ionosphere. The void was not filled by the ionospheric ions as they did not drift (or very slowly drifted) downstream. The dynamical pressure at the boundary was negligible because the plasma did not move perpendicular to the field. The void was maintained by the balance between low thermal pressure of the order of 5 pPa and the magnetic field pressure. The void was clearly identified in the MEX measurement (Extended Data Fig. 3a) at 09:00, matching the simulations. The lunar wake, which had a near-parallel IMF, formed by a similar mechanism^7^.

The planetary ions dynamics is driven by the **E** × **B** drift. **E** is dominated by the convective field as the ambipolar field is negligible at such altitudes. In the MSE frame, *B*_*z*_ = 0, *E*_*x*_ = *E*_*y*_ = 0 and *E*_*z*_ = *V*_0_*B*_*y*_. The components of the drift velocity **E** × **B** (*θ* is negative here) are as follows:

$$\begin{array}{ll}{V}_{x}\,=-{V}_{0}{\sin }^{2}\theta  & ({\rm{few}}\,{\rm{km}}\,{{\rm{s}}}^{-1})\\ {V}_{y}\,=-{V}_{0}\sin \theta \,\cos \theta  & ({\rm{tens}}\,{\rm{of}}\,{\rm{km}}\,{{\rm{s}}}^{-1})\\ {V}_{z}\,=0.\end{array}$$

The velocity parallel to the magnetic field (${V}_{\parallel }^{i}$) corresponds to the energy gained in the ambipolar field and was about one to tens of km s^−1^. Therefore, the planetary ions experienced very slow downstream convection while drifting faster in the **E** × **B** direction (predominantly in the y direction). This motion created a huge plume of ionospheric ions extending up to a distance of 15 Martian radii (Fig. 1). The planetary ions on the dayside accelerated by the ambipolar field moved far upstream along the magnetic field lines, forming an upstream cloud. As the solar wind reached down to low altitudes where the ionospheric densities were high, it transferred energy more effectively to the denser part of the ionosphere than in the nominal case, thus enhancing ion escape. Figure 2 shows the basic domains of a degenerate, induced magnetosphere and the near-Mars environment for a near-parallel IMF.

Our current model does not incorporate the crustal fields, which were primarily on the dayside. Nevertheless, during the time period studied, as the spacecraft traversed from the dayside to the nightside, the absence of crustal fields is unlikely to have significantly impacted the model’s alignment with measurements. The primary region where the solar wind interacted with the crustal fields was below 800 km (refs. ^8,9^), approximately equivalent to two pixels in our model. The omission of the crustal fields is not expected to alter the overarching dynamics of the solar wind’s interaction with the planet or the global structure of the degenerate, induced magnetosphere, but this remains a topic for future investigations.

Another significant subject for future studies is the role of magnetic field fluctuations in the ion dynamics. As shown by Luhmann et al.^10^, there was strong turbulence in the magnetic field in the interaction region when the cone angle was small. Similar strong turbulence was observed in the MAVEN data (Extended Data Fig. 2f,g). The transverse component of the fluctuating field accelerated the planetary ions. Simulations have shown that planetary ions can reach energies of up to 800 eV under Venus conditions. Thus, degenerate, induced magnetospheres are natural laboratories for studying wave–particle interactions.

Degenerate, induced magnetospheres are complex and not yet explored objects. Although they are relatively rare in the Solar System, they could be the nominal case for unmagnetized exoplanets in regions close to their parent star where the aberrated stellar wind happens to align with the magnetic field in the nominal Parker spiral direction.

## Methods

### Introduction

Unmagnetized bodies with sufficiently dense ionospheres, such as Mars and Venus, can be considered as conductors immersed in the moving solar wind plasma with velocity **V**_0_ carrying the frozen-in IMF **B**_0_. In the frame of reference of the conductor, the electromagnetic field comprises two components: **B**_0_ and the convective electric field **E** = −**V**_0_ × **B**_0_. The convective field results in a potential difference across the conductor, which, in turn, results in induction currents flowing through the conductor (unipolar induction). The magnetic fields associated with the induction currents cancel (or reduce because of finite conductivity) the magnetic field inside the ionosphere (Lenz’s law). Above the ionosphere, the induced fields act on the solar wind plasma by deviating it. Thus, a void called an induced magnetosphere is created. The magnetic pressure of the induced fields balances the dynamical and thermal pressures of the hot magnetosheath plasma. Under nominal conditions, the Parker angle at Mars is about 57° (ref. ^2^), and the mechanism above operates. However, in some rare cases, the cone angle, which is the angle between the solar wind velocity and the IMF, can be very low, below about 10°. For example, this occurred during approximately 2% of all MAVEN orbits from 2014 to 2019. In this case, the convective electric field responsible for generating the induction currents weakens, or, in other words, the magnetic flux through the conductive ionosphere responsible for the electromotive force significantly reduces, so that the mechanism described above does not operate any more. When the interaction of unmagnetized bodies with the solar wind occurs for very small cone angles so that the convective electric field is very weak, we say that the induced magnetosphere is degenerate. Dubinin et al.^3^ noted this very peculiar case for the interaction at Venus and also identified the interaction as a degenerate, induced magnetosphere. This type of interaction has recently attracted attention^11–13^. We use the term degenerate, induced magnetosphere to emphasize the fundamental difference in the structure and physics of such interaction.

Earlier studies of the solar wind’s interaction with Venus for small cone angles, which utilized Pioneer Venus Orbiter measurements, focused on magnetosheath turbulence and wave–particle interactions that resulted in planetary-ion pickup^14^. The structure and morphology of the interaction region have received less attention. In general, the electrodynamics of degenerate magnetospheres are not well understood, and the question is open as to what, if anything, serves as a solar wind obstacle. From measurements at Mars and Venus, it is still not clear whether or not a magnetosphere-like void or, in other words, a magnetic barrier, forms for very small cone angles.

De Zeeuw et al.^15^ used a two-dimensional magnetohydrodynamics model to simulate the interaction between the solar wind and Venus during a radial IMF and found that a magnetotail was not induced. Using data from the magnetometer onboard Venus Express, Zhang et al.^11^ conducted a case study in which the cone angle was 11° and noted the absence of a solar wind void, referred to as a ‘magnetic barrier’ in their terminology. Dubinin et al.^3^ analysed combined magnetic field and particle data from Venus Express and highlighted that ‘spatially varying IMF across the planet will produce a magnetic field which looks like the field of a degenerated dipole with its axis aligned with the solar wind flow’ so that a magnetic barrier forms. Chang et al.^12^ performed a statistical study of all available Venus Express data and identified 13 cases when the cone angle was below 20°. Those authors emphasized the role of the magnetic field tension ((**B** ⋅ ∇)**B**/*μ*_0_) in deviating the solar wind plasma. This tension contributed to the existence of a ‘weak’ magnetic barrier that could balance the solar wind’s dynamical pressure, although it is not clear how, as this term was very small. Fowler et al.^13^ analysed in detail low-altitude observations (down to 250 km) of the solar wind at small solar zenith angles (20°–30°) made by the MAVEN spacecraft. They concluded that this case corresponds to a small cone angle, although MAVEN, being in a low-altitude orbit, did not reach the undisturbed solar wind. A pressure balance analysis indicated that the magnetic pressure in the region matched the estimated upstream dynamic pressure of the solar wind. Fowler et al.^13^ suggested that a magnetic barrier formed in the deep ionosphere.

Pioneer Venus Orbiter observed a quasi-parallel bow shock at Venus, which extended to high solar zenith angles, resulting in intense turbulence, wave–particle interactions and heating of planetary ions on the dayside^10^. Fowler et al.^13^ reported similar heating at Mars. Ions propagate upstream along parallel magnetic field lines, resulting in erosion of the ionospheric density, as seen in hybrid simulations of Venus by Omidi et al.^16^. Masunaga et al.^17^ claimed that the total escape rate does not depend on the cone angle, but their focus was solely on the downtail target area.

Degenerate, induced magnetospheres are open systems, meaning that the solar wind can reach the ionosphere or even the collisional boundary^13^. This enables the transfer of energy, associated with both waves and direct kinetic energy of the solar wind particles, from the upstream region to the ionosphere, which could be another ionization source^18^. All these features make a degenerate, induced magnetosphere a distinctive mode of solar wind interactions that warrants dedicated detailed studies. Degenerate, induced magnetospheres are also common for unmagnetized exoplanets close to their parent stars, where the magnetic field tends to be nearly parallel to the stellar wind.

### Observations by MAVEN and MEX

**Extended Data Fig. 1:**
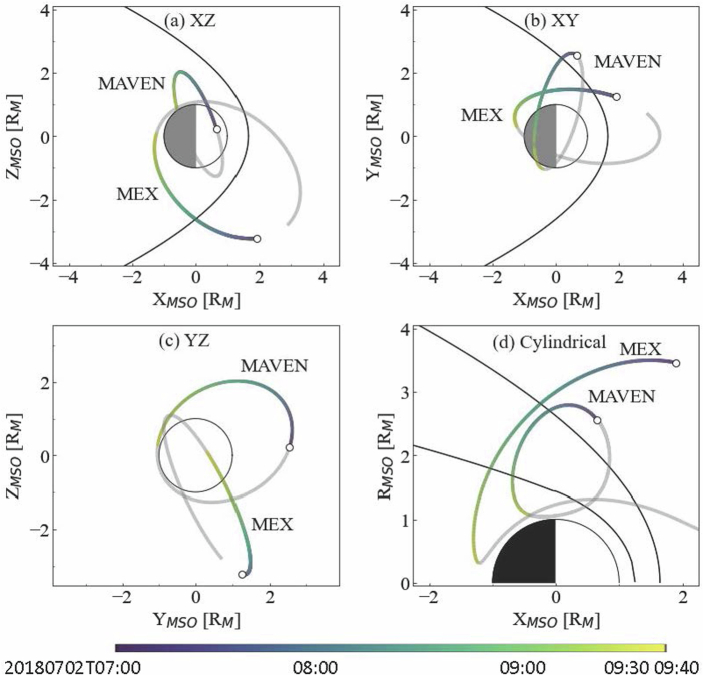
Trajectories of MAVEN and MEX in the MSO coordinate system (the *x*-axis is directed to the sun, the *y*-axis is in the Mars orbital plane, perpendicular to the *x*-axis and opposite to Mars’ motion, and the *z*-axis completes the right-handed coordinate system). The colored inbound part of the orbits represents the time interval of this study. The circles show the starting points of the observations. The black lines show the nominal bow shock and induced magnetosphere boundary (IMB) locations as derived by Trotignon et al.^28^. The colorbar at the bottom denotes the time.

**Extended Data Fig. 2:**
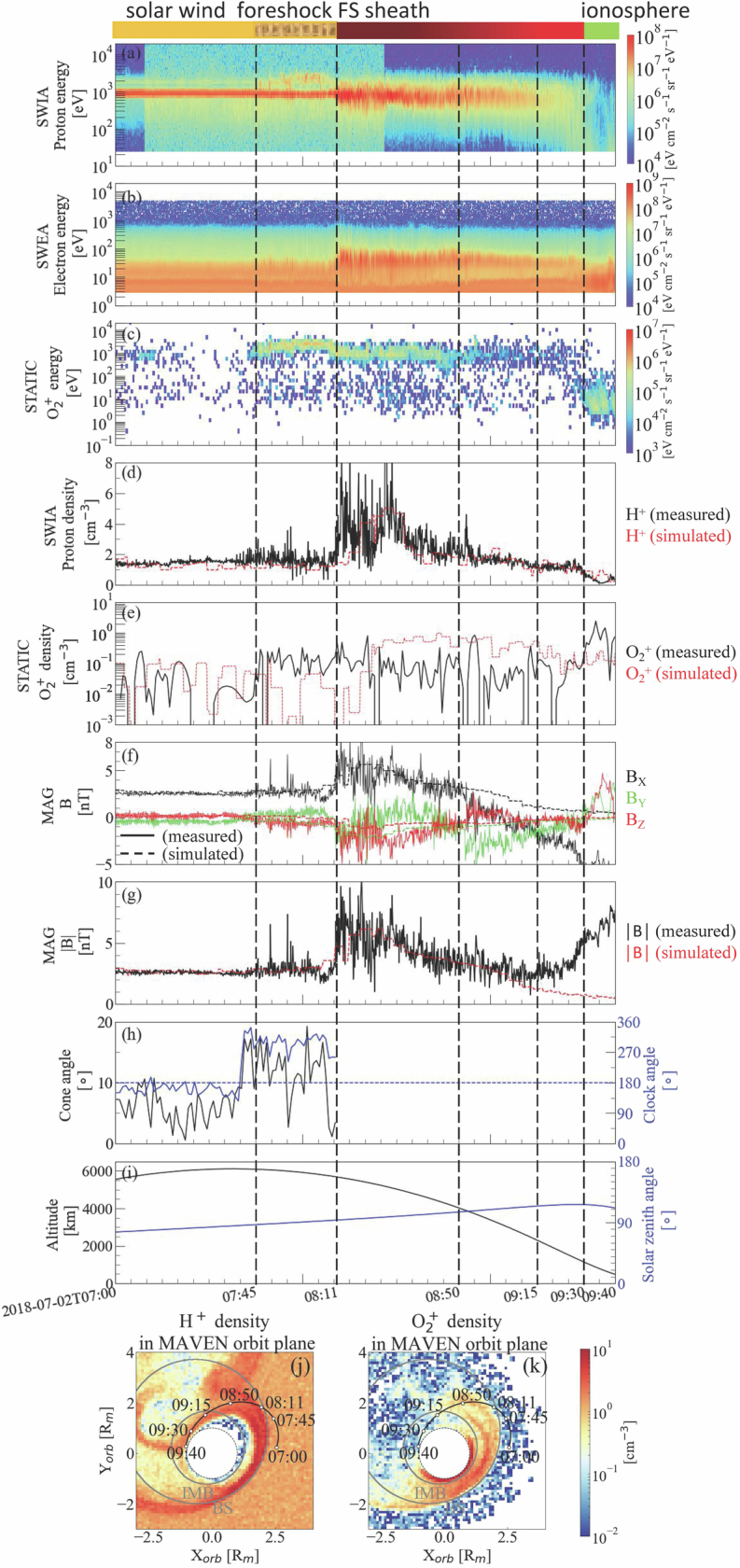
MAVEN observation and comparison with simulation. The panels show (a) SWIA proton energy spectrum; (b) SWEA electron energy spectrum; (c) STATIC ${{\rm{O}}}_{2}^{+}$ energy spectrum; (d) SWIA and simulated (dashed red line) proton density; (e) STATIC and simulated (dashed red line) ${{\rm{O}}}_{2}^{+}$ density; (f) MAG and simulated (dashed lines) magnetic field vector; (g) MAG and simulated (dashed red line) magnetic field magnitude; (h) cone angle between the solar wind velocity and the interplanetary magnetic field (IMF), and clock angle is the IMF counterclockwise angle from + *y*-axis in the MSO coordinate; (i) spacecraft altitude and solar zenith angle. The vertical dashed lines mark out the time stamps for the comparison between the measurements and model. Panels at the bottom show the simulated proton density (j) and ${{\rm{O}}}_{2}^{+}$ density (k) in the MAVEN orbit plane. The black lines are the MAVEN trajectories. The grey lines are the nominal bow shock and IMB^28^ projected in MAVEN orbit plane. The colorbar at the top denotes different plasma domains identified from the measurements. No IMB is identified.

**Extended Data Fig. 3:**
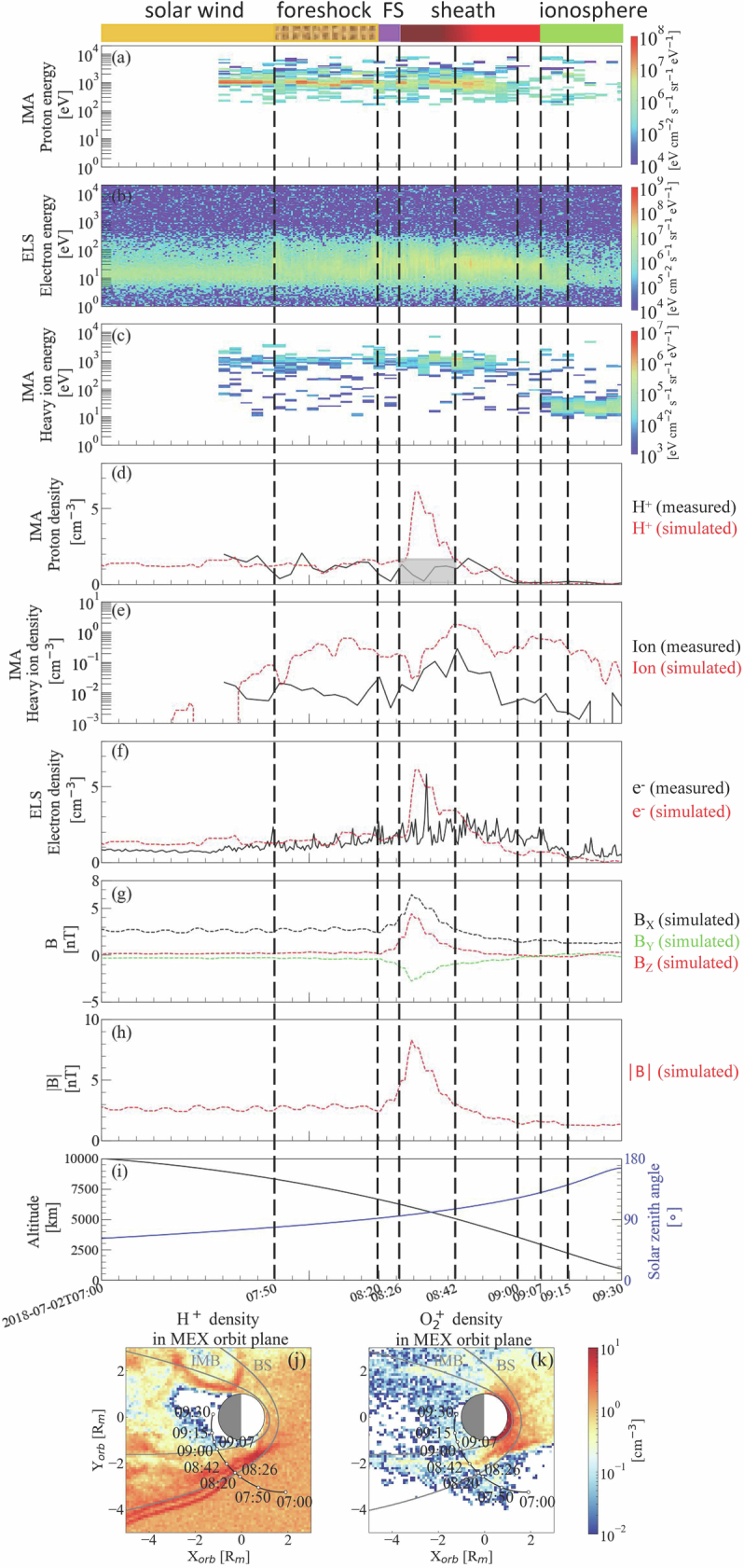
MEX observation and comparison with simulation. The panels show (a) IMA proton energy spectrum; (b) ELS electron energy spectrum; (c) IMA heavy ion energy spectrum; (d) IMA and simulated (dashed red line) proton density; (e) IMA and simulated (dashed red line) heavy ion density; (f) ELS and simulated (dashed red line) electron density; (g) simulated magnetic field vector; (h) simulated magnetic field magnitude; (i) spacecraft altitude and solar zenith angle. The vertical dashed lines mark out the time stamps for the comparison between measurements and model. Panels at the bottom show the simulated proton density (j) and ${{\rm{O}}}_{2}^{+}$ density (k) in the MEX orbit plane. The black lines are the MEX trajectories. The grey lines are the nominal bow shock and IMB^28^ projected in MEX orbit plane. The grey block in (d) indicates the solar wind obscured. The colorbar at the top defines the regions identified from the measurement. No IMB is identified.

This study is based on simultaneous observations (Extended Data Fig. 1) by MAVEN (Extended Data Fig. 2) and MEX (Extended Data Fig. 3) from 07:00 to 09:40 utc on 2 July 2018. We utilized measurements made by the solar wind ion analyser^19^, the solar wind electron analyser^20^, the suprathermal and thermal ion composition instrument^21^ and the magnetometer^22^ onboard MAVEN. We also used measurements made by the ion mass analyser^5^ and the electron spectrometer^5^ onboard MEX. The cone angle was 4° as calculated from the solar wind ion analyser and magnetometer data in the undisturbed solar wind (Extended Data Fig. 2h). The other upstream parameters, detailed in Extended Data Table 1, are typical for Mars. The upstream parameters in the table were computed by averaging MAVEN observations over time from 07:00 to 07:45 when the spacecraft was in the undisturbed solar wind, as can be seen in Extended Data Fig. 2. The cone angle of 4° that we got from the averaged magnetic field and velocity values is smaller than the cone angle of 7° averaged from the instantaneous values. This ambiguity was unavoidable, as we had to pick only one set of upstream conditions to represent the observed time-variable upstream conditions for our simulations. We chose only the inbound part of the orbits for this study because the solar wind conditions could have changed while the spacecraft was inside the interaction region. MAVEN collected data from upstream at the terminator. It had an apoapsis altitude of approximately 6,100 km to the terminator’s ionosphere and reached a periapsis altitude of approximately 165 km (Extended Data Fig. 1). MEX sampled from further upstream on the dayside, with an apoapsis altitude of approximately 10,000 km. It extended deeper into the magnetotail and reached a periapsis altitude of approximately 370 km (Extended Data Fig. 1).

MAVEN’s measurements relevant for the analysis start from the undisturbed solar wind. At 07:45, the spacecraft entered the foreshock region, a disturbed region upstream of a shock-like boundary (see more discussion below). This region was identified by the strong fluctuations in the magnetic field (Extended Data Fig. 2f,g) and the extra populations of ions with an energy higher than the solar wind energy (Extended Data Fig. 2a). The latter were planetary ions and visible in Extended Data Fig. 2c, but note that the solar wind ion analyser does not resolve masses. These ions were at considerable distances from the shock crossing at 08:11 (Extended Data Fig. 2c,e). By applying the magnetic coplanarity theorem^23^, we obtained a shock normal angle of 46°. Thus, the shock, if it is indeed a shock, was quasi-perpendicular, as expected, in the flank. It was probably a weak shock, as revealed by the insignificant proton and electron heating, and the Mach number barely changed on passing through this weak shock (from 8 to 7), but the exact nature of such shocks for degenerate, induced magnetospheres requires further investigation. Hence, we refer to it as a flank shock in this paper, although no clear induced-magnetosphere boundary has been identified, because there was no sharp enhancement of the magnetic field accompanied by an increase of planetary ions and cold electrons. If the shock is confirmed in future studies, the nature of the obstacle creating it is a puzzle. During the period 07:45–08:50, MAVEN was moving through a structure specific for degenerate, induced magnetospheres, a cross-flow plume of heavy ions, which overlapped with the magnetosheath-like region identified by the weakly heated protons. The magnetic field exhibited strong fluctuations, a factor of four in magnitude, that could also be important for understanding the physics of the flank shock. The exact nature of this domain is not clear either, as the conventional features of an induced magnetosphere and bow shock were not observed. From 08:50 to 9:30, the heavy-ion fluxes slowly decreased. At 09:30, MAVEN entered the ionosphere, as was identified by the presence of a low-energy planetary-ion population. The solar wind persisted until it reached the ionosphere (Extended Data Fig. 2d). For comparison with simulations, we also present macroscopic parameters obtained by integrating the measured differential fluxes, the proton and ${{\rm{O}}}_{2}^{+}$ densities, as well as the magnetic field.

Extended Data Fig. 3 shows observations from MEX, including the energy spectra and densities of protons, heavy ions and electrons. Like MAVEN, MEX also captured features of a strong foreshock between 07:50 and 08:20 (Extended Data Fig. 3b), high fluxes of heavy ions in the sheath region (Extended Data Fig. 3c) and no typical signatures of a crossing of the induced-magnetosphere boundary. The proton, heavy-ion and electron densities in Extended Data Fig. 3d–f were derived by integrating the respective differential fluxes. Although there was reasonable agreement between the proton and electron densities (the heavy ions contributed very little) when MEX was in the solar wind, the densities were significantly different from 08:26 to 08:42. This interval corresponds to when MEX crossed the flank shock and entered the magnetosheath-like structure. The discrepancy could be attributed to the shock being weak. As MEX passed through this weak shock, the solar wind maintained its supersonic speed but underwent a directional change and became partially obscured by the spacecraft structure, owing to how the ion mass analyser was installed on MEX. As the shock was weak, there was no substantial heating. The ion mass analyser covered only a fraction of the distribution function, leading to an underestimation of the proton density. The electron density measurements, however, are reliable because of the broader electron distribution function.

The dashed lines in Extended Data Figs. 2 and 3 represent the results from the hybrid model, which were compared with observations.

### Hybrid simulations

Using the observed upstream parameters from MAVEN (Extended Data Table 1), we performed a hybrid simulation of the relevant interaction. The model is described in more detail in Zhang et al.^24,25^, Holmstrom^26^ and references therein. In the model, electrons are treated as an inertialess charge-neutralizing fluid, whereas ions are treated as particles (grouped together as macroparticles). The ion motion is determined by the fields obtained from a generalized Ohm’s law. The model incorporates two solar wind species (H^+^ and He^++^) and three planetary species (${{\rm{O}}}^{+},{{\rm{O}}}_{2}^{+}$ and ${{\rm{CO}}}_{2}^{+}$). The ionosphere is represented by the upflux of ionospheric ions from its inner boundary. The heavy ions are produced on the dayside from the obstacle boundary, with an initial velocity drawn from a Maxwellian distribution corresponding to a temperature of 200 K. The ion upflux decays from the subsolar point to the terminator by the cosine of the solar zenith angle. The ion upflux is a free parameter in our model.

We used MAVEN’s upstream observations as input in the model. We ran different simulations with different ion upfluxes. We thereafter chose the run that best matched the observations. The total ion upflux we used was 4.8 × 10^25^ s^−1^. The model was configured in the MSE frame, with the the *x* axis antiparallel to the solar wind velocity, the *z* axis aligned with the solar wind convective electric field −**V** × **B** and the *y* axis completing the right-handed coordinate system. The magnetic field was stored on a uniform grid with a spatial resolution of 350 km. The inner boundary was set at 170 km altitude above Mars, approximately corresponding to the exobase altitude. Ions inside the inner boundary were removed from the simulation. We solved a magnetic diffusion equation inside the inner boundary. Crustal fields were not included in the model.

**Extended Data Fig. 4:**
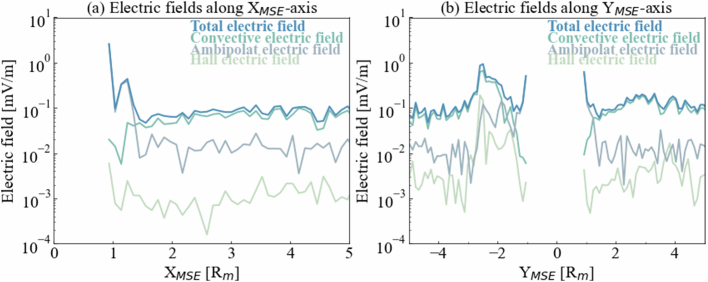
Simulation results of the electric fields, including the total electric field and the three terms. the convective electric field, the ambipolar electric field and the Hall electric field, along *X*_*MSE*_-axis and *Y*_*MSE*_-axis.

Extended Data Fig. 4 illustrates that the convective electric field predominated at high altitudes, whereas the ambipolar field was dominant near the planet, at altitudes of several hundreds of kilometres.

### Comparison of the simulations and measurements

The model results are compared both to MAVEN and MEX measurements made along their trajectories (dashed lines in Extended Data Figs. 2d–g and 3d–h). An overview of the proton density and ${{\rm{O}}}_{2}^{+}$ density distributions in their orbit planes is shown in Extended Data Figs. 2j–k and 3j–k. In general, the simulation and measurements are in good agreement.

MAVEN’s orbit was almost in the terminator plane. The agreement can be seen in the proton density (Extended Data Fig. 2d) and magnetic field (Extended Data Fig. 2g). At 7:45, the oscillations of the magnetic field increased in the foreshock region, coinciding with the observations of planetary ions. Around 8:11, an abrupt rise in the magnetic field magnitude and proton density indicates a flank shock crossing, which is evident in the model as well (Extended Data Fig. 2d,j). The prominent ${{\rm{O}}}_{2}^{+}$ flux from 7:45 to 8:50, visible in Extended Data Fig. 2k, indicates the presence of planetary ions in the magnetosheath-like structure. The delayed increase in ${{\rm{O}}}_{2}^{+}$ density in the model compared to measurements may stem from the spacecraft orbiting along the edge of the larger ${{\rm{O}}}_{2}^{+}$ flux region, rendering the model flux sensitive to the exact location. Around 9:15, MAVEN observed a decrease in the proton and ${{\rm{O}}}_{2}^{+}$ densities. It entered the nightside and encountered an ${{\rm{O}}}_{2}^{+}$ density gap, as seen in the model (Extended Data Fig. 2j,k).

The comparison to MEX data is more difficult because of complications in the proton observations and the absence of magnetic field measurements. The flank shock was crossed at 08:20, which was marked by a significant increase in the electron energy spectrum (Extended Data Fig. 3b), although this effect was not mirrored in the proton density (Extended Data Fig. 3d) for the reasons outlined above. The modelled shock crossing was about 5 min late, possibly because the shock was highly dynamic, and therefore, the precise location may not match exactly. The modelled electron density agrees with the observations (Extended Data Fig. 3f). At 09:00, the proton fluxes vanished (Extended Data Fig. 3a), as MEX entered the proton void in agreement with the simulations (Extended Data Fig. 3j). The heavy ions became apparent (Extended Data Fig. 3c,e,k) even before the foreshock crossing at 07:50. The overall variability of heavy ions between observations and simulation (Extended Data Fig. 3e) aligns well, although the absolute values do not match perfectly. As it was difficult to reproduce all the details of the ionosphere in the model, we did not expect to achieve a quantity match between observations and models, instead we expected a quality match. The peak in the heavy-ion density (Extended Data Fig. 3e) at around 08:42, which is associated with the crossing of a tailward flow of heavy ions aligned with the local magnetic field, is well reproduced (Extended Data Fig. 3k). At 09:07, MEX entered the ionosphere, and cold ions became visible, as they did for MAVEN. From 09:15 onward, MEX was in the eclipse, as the photoelectron fluxes disappeared.

## Online content

Any methods, additional references, Nature Portfolio reporting summaries, source data, extended data, supplementary information, acknowledgements, peer review information; details of author contributions and competing interests; and statements of data and code availability are available at 10.1038/s41586-024-07959-z.

## Figures and Tables

**Extended Data Table 1:**
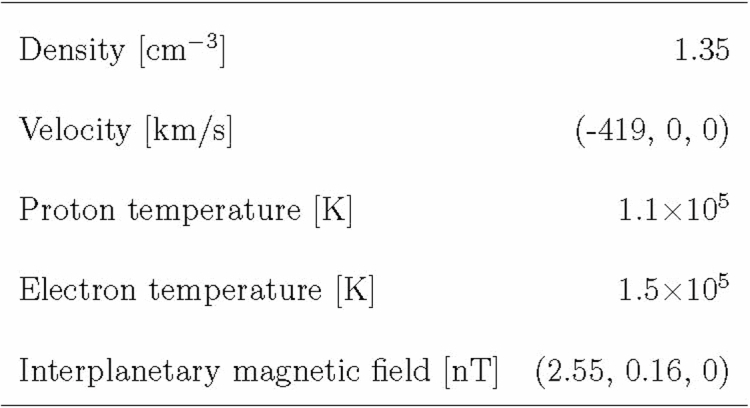
Upstream solar wind parameters on 2 July 2018 estimated from MAVEN observations in the MSE coordinate system (the *x*-axis is antiparallel to the solar wind velocity, the *z*-axis is aligned with the solar wind convective electric field −**V** × **B**, and the *y*-axis completes the right-handed coordinate system)

## Data Availability

The MEX data used in this work are available from the Science Data Centre of the European Space Astronomy Centre (https://archives.esac.esa.int/psa/ftp/MARS-EXPRESS/ASPERA-3/). The MAVEN data used in this work are available both from the NASA Planetary Data System (https://pds.nasa.gov/) and from the MAVEN Science Data Center (https://lasp.colorado.edu/maven/sdc/public/). Ion densities provided by the the suprathermal and thermal ion composition instrument onboard MAVEN are available from the Space Physics Environment Data Analysis Software (https://spedas.org/blog/).

## Code availability

The software used in this work is FLASH v.4.2, which was in part developed by the Flash Center for Computational Science at the University of Chicago^27^.
